# Supplementary figures and images for: Mathematical measures of societal polarisation
Source: PLoS One. 2022 Oct 4;17(10):e0275283. doi: 10.1371/journal.pone.0275283 (PMC9531819; doi:10.1371/journal.pone.0275283)

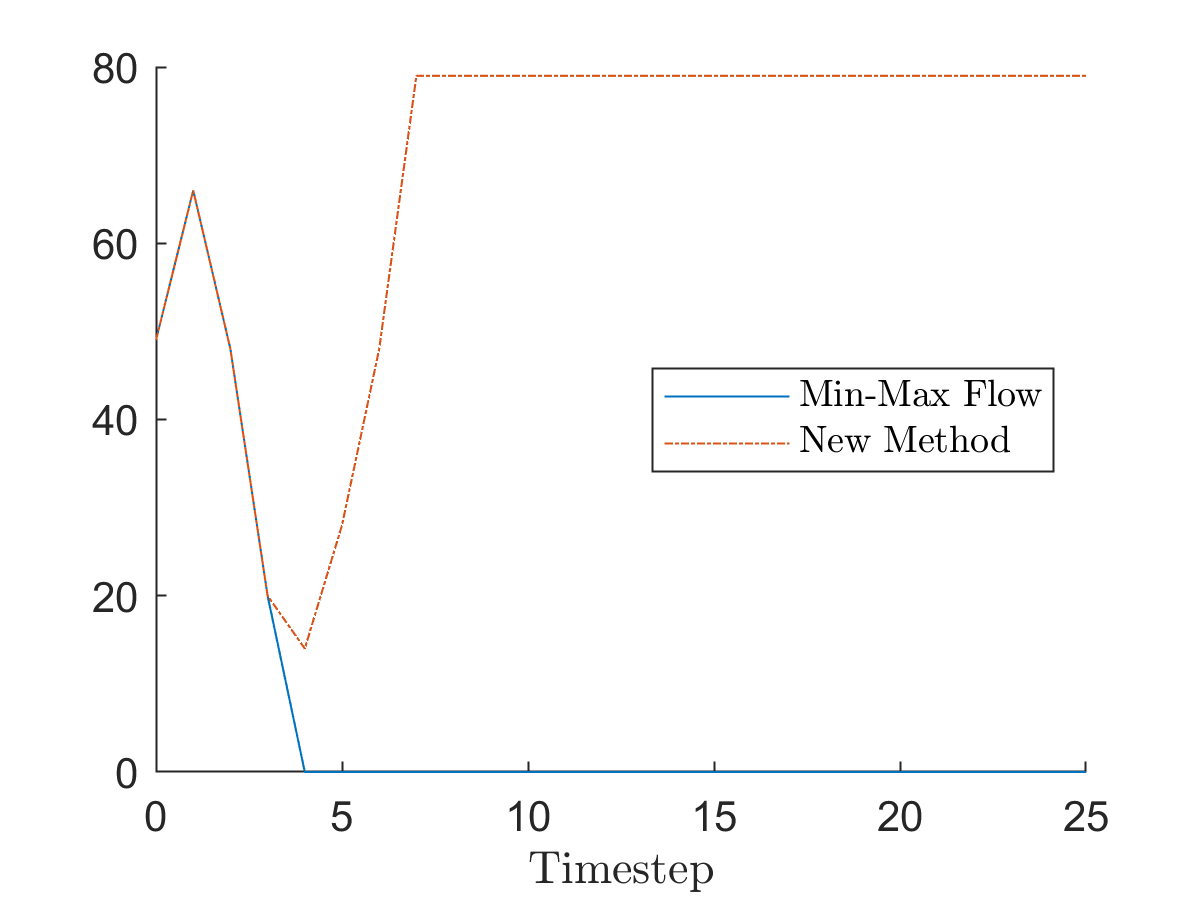

Supplement: S1 Fig — (TIF) [file pone.0275283.s001.tif]
