# Supplementary material for: Mathematical measures of societal polarisation
Source: PLoS One. 2022 Oct 4;17(10):e0275283. doi: 10.1371/journal.pone.0275283 (PMC9531819; doi:10.1371/journal.pone.0275283)
Supplement: S1 File — (PDF) [file pone.0275283.s002.pdf]

S1 Appendix. Basics of Graph Theory. A graph G consists of a set of vectors, which we will call nodes, V where $v_i \in V$ is the ith node of G and a set of edges E where $e_{ij} \in E$ means that $v_i, v_j \in V$ are connected in G. We can define a path P in G to be a set of edges in G such that if you begin at v_i and follow the edges in P you will finish at v_j such that $P = \{e_{i1}, e_{12}, \ldots, e_{nj}\}$. A component is defined as the set of nodes $V_i \subset V$ in G such that paths exist between all nodes of V_i, but no path exists to nodes outside of V_i. A graph G is disjoint if G has more than one component; likewise, G is 'connected' when only one component exists. A directed graph, digraph, is a graph G that has its edges E have a direction, i.e. v_1 connects to v_2, but v_2 doesn't necessarily connect back to v_1. A weighted graph is where for a graph G there exists a function $f : E \to \mathbb{R}$.

S2 Appendix.

Theorem 1. *Let A be a square block diagonal matrix consisting of m square matrices of ones J_{n_i} where n_i is the dimension of the matrix of ones i, and $i = 1, \ldots, m$. Then the spectral radius $\rho(A)$ is equal to the dimension of the largest unit matrix in A.*

Proof. Since A is block diagonal, the eigenvalues of A are the eigenvalues of $J_{n_1}, \ldots, J_{n_m}$. We know that for a general square matrix of ones J_k [1] its characteristic equation is

$$0 = (k - \lambda)\,\lambda^{k-1}.$$

The dimensional values $n_1, \ldots, n_m$ are all eigenvalues of their respective matrix including multiple eigenvalues equal to 0. Then $n_1, \ldots, n_m$ must be eigenvalues of A, the largest of which is $\rho(A)$, which is also the dimension of the largest matrix of ones in A.

S3 Appendix. Alternate min-max flow for HK bounded confidence. To simplify the calculation of the min-max flow rate for the HK bounded confidence model, one might consider the following method:

1. For every agent in a simulation, count the number of agents within ϵ of the agent's opinion.
2. Minimise over those agent counts.

Such a method counts the degree of each agent if you were to develop an adjacency matrix at that specific time in the simulation. Intuitively the method should be identical to min-max flow, but it produces different results when a simulation enters polarisation (see S1 Fig for an example). This method converges to the smallest cluster size instead of reaching zero. We thus consider this method distinct from the min-max flow method.

S4 Appendix. Derivation of the KLD of two normal distributions. Let $X \sim \mathcal{N}(\mu_i, \sigma_i^2)$ and $Y \sim \mathcal{N}(\mu_j, \sigma_j^2)$, and $f(x)$ and $g(y)$ describe the probability density

function for X and Y then

$$
\begin{aligned}
\mathrm{KLD}(X,Y) &= \int_{-\infty}^{\infty} f(x)\log\left(\frac{f(x)}{g(x)}\right)\,\mathrm{d}x \\
&= \int_{-\infty}^{\infty} f(x)\left[\log\left(\frac{\sigma_j\sqrt{2\pi}}{\sigma_i\sqrt{2\pi}}e^{-\frac{(x-\mu_i)^2}{2\sigma_i^2}+\frac{(x-\mu_j)^2}{2\sigma_j^2}}\right)\right]\,\mathrm{d}x \\
&= \int_{-\infty}^{\infty} f(x)\left[\log\left(\frac{\sigma_j}{\sigma_i}\right)-\frac{(x-\mu_i)^2}{2\sigma_i^2}+\frac{(x-\mu_j)^2}{2\sigma_j^2}\right]\,\mathrm{d}x \\
&= \log\left(\frac{\sigma_j}{\sigma_i}\right)\int_{-\infty}^{\infty} f(x)\,\mathrm{d}x-\frac{1}{2\sigma_i^2}\int_{-\infty}^{\infty}(x-\mu_i)^2 f(x)\mathrm{d}x \\
&\qquad\qquad\qquad\qquad+\frac{1}{2\sigma_j^2}\int_{-\infty}^{\infty}(x-\mu_j)^2 f(x)\mathrm{d}x \\
&= \log\left(\frac{\sigma_j}{\sigma_i}\right)-\frac{1}{2\sigma_i^2}\mathrm{Var}\left(X\right)+\frac{1}{2\sigma_j^2}\int_{-\infty}^{\infty}\left(x^2-2\mu_j x+\mu_j^2\right)f(x)\,\mathrm{d}x \\
&= \log\left(\frac{\sigma_j}{\sigma_i}\right)-\frac{1}{2}+\frac{1}{2\sigma_j^2}\int_{-\infty}^{\infty}\left((x-\mu_i)^2+2x\left(\mu_i-\mu_j\right)+\mu_j^2-\mu_i^2\right)f(x)\,\mathrm{d}x \\
&= \log\left(\frac{\sigma_j}{\sigma_i}\right)-\frac{1}{2}+\frac{1}{2\sigma_j^2}\left[\int_{-\infty}^{\infty}(x-\mu_i)^2 f(x)\,\mathrm{d}x+2\left(\mu_i-\mu_j\right)\int_{-\infty}^{\infty}xf(x)\,\mathrm{d}x\right. \\
&\qquad\qquad\qquad\qquad\left.+\left(\mu_j^2-\mu_i^2\right)\int_{-\infty}^{\infty}f(x)\,\mathrm{d}x\right] \\
&= \log\left(\frac{\sigma_j}{\sigma_i}\right)-\frac{1}{2}+\frac{1}{2\sigma_j^2}\left[\mathrm{Var}\left(X\right)+2\left(\mu_i-\mu_j\right)E\left(X\right)+\mu_j^2-\mu_i^2\right] \\
&= \log\left(\frac{\sigma_j}{\sigma_i}\right)-\frac{1}{2}+\frac{\sigma_i^2+2\left(\mu_i-\mu_j\right)\mu_i+\mu_j^2-\mu_i^2}{2\sigma_j^2} \\
&= \log\left(\frac{\sigma_j}{\sigma_i}\right)+\frac{\sigma_i^2+\left(\mu_i-\mu_j\right)^2}{2\sigma_j^2}-\frac{1}{2}
\end{aligned}
$$

S5 Appendix. Derivation of the H-distance of two uniform distributions. Let x_1 and x_2 be the centers of two uniform distributions $f(x)$ and $g(x)$ both with width 2ϵ and, without loss of generality, let $x_1 > x_2$. The Hellinger distance is

$$H^2(f,g) = 1 - \int_{-\infty}^{\infty}\sqrt{f(x)g(x)}\,\mathrm{d}x.$$

There is two distinct cases for the Hellinger affinity. First is when there is no overlap, i.e. $x_1 - x_2 > 2\epsilon$, between f and g which means that the Hellinger affinity is zero and hence

$$\int_{-\infty}^{\infty}\sqrt{f(x)g(x)}\,\mathrm{d}x = 0,$$

therefore

$$H^2(f,g) = 1.$$

Second is when there is overlap, i.e. $x_1 - x_2 \leq 2\epsilon$, and the Hellinger affinity is non-zero. Specifically the Hellinger affinity will be the area of the overlap which is

$$\int_{-\infty}^{\infty}\sqrt{f(x)g(x)}\,\mathrm{d}x = \frac{2\epsilon + x_2 - x_1}{2\epsilon},$$

therefore,

$$H^2(f,g) = \frac{x_1 - x_2}{2\epsilon}.$$

We can conclude that

$$H^2(f,g) = \begin{cases} \frac{x_1 - x_2}{2\epsilon} & \text{if } x_1 - x_2 \leq 2\epsilon \\ 1 & \text{if } x_1 - x_2 > 2\epsilon \end{cases}.$$

S6 Appendix. Estimating cluster count from exponential, mean KLD **growth.** Consider a Martins simulation that has reached steady-state, let Ω be the set of all agents in the simulation, and the simulation has divided into ψ separate opinion clusters such that

$$\Omega = \bigcup_{k=1}^{\psi} A_k,$$

where A_k is a set of agents in the kth opinion cluster such that

$$A_k \cap A_l = \emptyset, \ \forall k, l$$
$$|A_k| = |A_l|, \ \forall k, l$$

In steady-state, all agents in a Martins simulations have $\sigma \to 0$. Then, according to Eq 5, agents will only achieve a $p^* = 1$ when $x_i = x_j$ i.e. when two agent are in the same cluster. If $x_i \neq x_j$ i.e. when two agent are in the different clusters, then, with $\sigma \to 0$, $p^* = 0$. Also, from Eq 5, when $p* = 1$, agents will halve their σ^2, which means that Eq 7 will double for a select proportion of agent pairs in the simulation, but not for all agent pairs. Meaning that $\overline{\text{KLD}}$ will proportionally grow by a fixed amount a after a single $p^* = 1$ interaction

$$\frac{\overline{\text{KLD}}(t+s)}{\overline{\text{KLD}}(t)} = a, \tag{9}$$

where t is an arbitrary number of interactions after the simulation has reached steady-state and s is the number of interactions until a $p^* = 1$ interaction occurs.

In a single $p^* = 1$ interaction, two agents will halve their 'variance' (uncertainty squared), doubling the KLD between those agents and every other agent in the simulation. All other pairings will maintain the same KLD. The proportional growth of $\overline{\text{KLD}}$, a, is dependent only on the pairwise agent's KLD where KLD $\neq 0$. We refer to agent pairings that have a KLD $= 0$ as non-contributing and those with KLD > 0 as contributing. Therefore,

$$a = (1-q) + 2q = 1 + q$$

where q is the proportion of contributing pairings that double their KLD. Let n be the number of agents in the simulation and consider an agent inside a cluster, only $n(1 - 1/\psi)$ agents would generate contributing KLDs since agents inside the hypothetical agent's cluster would generate a KLD $= 0$. Since two agents will be interacting, we can double this agent count to get the total number KLDs that double from two opinions updating, resulting in $2n(1 - 1/\psi)$. The total number of contributing pairings will be the total number of possible pairings, n^2, minus the non-contributing pairings, i.e. pairings which pair agents from the same clusters, $\psi(n/\psi)^2$. Therefore the total number of contributing pairings is $n^2(1 - 1/\psi)$. It follow then that $q = 2/n$. Thus

$$a = \frac{n+2}{n},$$

and Eq 9 becomes

$$\frac{\overline{\mathrm{KLD}}(t+s)}{\overline{\mathrm{KLD}}(t)} = \frac{n+2}{n}. \tag{10}$$

Now consider a simulation which enters steady state after t_0 interactions, without loss of generality let $t_0 = 0$. Let s_1 be the number of interactions until the first $p^* = 1$ interaction occurs, from Eq 10 we know that

$$\frac{\overline{\mathrm{KLD}}(t_0+s_1)}{\overline{\mathrm{KLD}}(t_0)} = \frac{n+2}{n},$$
$$\overline{\mathrm{KLD}}(s_1) = \frac{n+2}{n} K_0,$$

where $K_0 = \overline{\mathrm{KLD}}(t_0)$. For the second $p^* = 1$ interaction s_2, using Eq 10 again, we can show

$$\frac{\overline{\mathrm{KLD}}(s_1+s_2)}{\overline{\mathrm{KLD}}(s_1)} = \frac{n+2}{n},$$
$$\overline{\mathrm{KLD}}(s_1+s_2) = \left(\frac{n+2}{n}\right)^2 K_0.$$

So for the general rth $p^* = 1$ interaction s_r we have

$$\overline{\mathrm{KLD}}\left(\sum_{i=1}^{r} s_i\right) = \left(\frac{n+2}{n}\right)^r K_0. \tag{11}$$

Let t be the total number of interaction after t_0, then

$$r = \frac{t}{\bar{s}},$$

where

$$\bar{s} = \frac{1}{r}\sum_{i=1}^{r} s_i.$$

Therefore Eq 11 becomes

$$\overline{\mathrm{KLD}}\left(t\right) = \left(\frac{n+2}{n}\right)^{t/\bar{s}} K_0, \tag{12}$$

and because

$$\frac{n+2}{n} > 1,$$

$\overline{\mathrm{KLD}}$ will grow exponentially in t.

The simulation chooses agents at random to interact, so $s_1, \ldots, s_r$ will be valued from the same random variable S. which will follow a geometric distribution with the probability of success equal to the probability of interaction being a $p^* = 1$ interaction, or the probability that for any $k \in 1, \ldots, \psi$ the simulation chooses two agents from a cluster A_k. Therefore the probability of success in S is $1/\psi$ which implies $E\left(S\right) = \psi$ and by extension $E\left(\bar{s}\right) = \psi$, hence $\bar{s}$ is an unbiased estimator or ψ. Substituting $E\left(\bar{s}\right) = \psi$ Eq 12 becomes

$$\overline{\mathrm{KLD}}\left(t\right) = \left(\frac{n+2}{n}\right)^{t/\psi} K_0. \tag{13}$$

Taking the log on both sides of Eq 13 gives us

$$\log\left(\overline{\mathrm{KLD}}\left(t\right)\right) = \log\left(K_0\right) + \frac{\log\left(n+2\right) - \log\left(n\right)}{\psi} t,$$

we can estimate the effective cluster count $\widehat{\psi}$ by fitting a linear model with slope m to simulation data $\log\left(\overline{\mathrm{KLD}}\left(t\right)\right)$ using a least squares estimation. The general expression for the effectively estimated cluster count is

$$\widehat{\psi} = \frac{\log\left(n+2\right) - \log\left(n\right)}{m}.$$

Reference

1. Stanley RP. Lemma 1.4. In: Algebraic Combinatorics. Springer-Verlag GmbH; 2013. p. 4. Available from: https://www.ebook.de/de/product/22972158/richard_p_stanley_algebraic_combinatorics.html.
